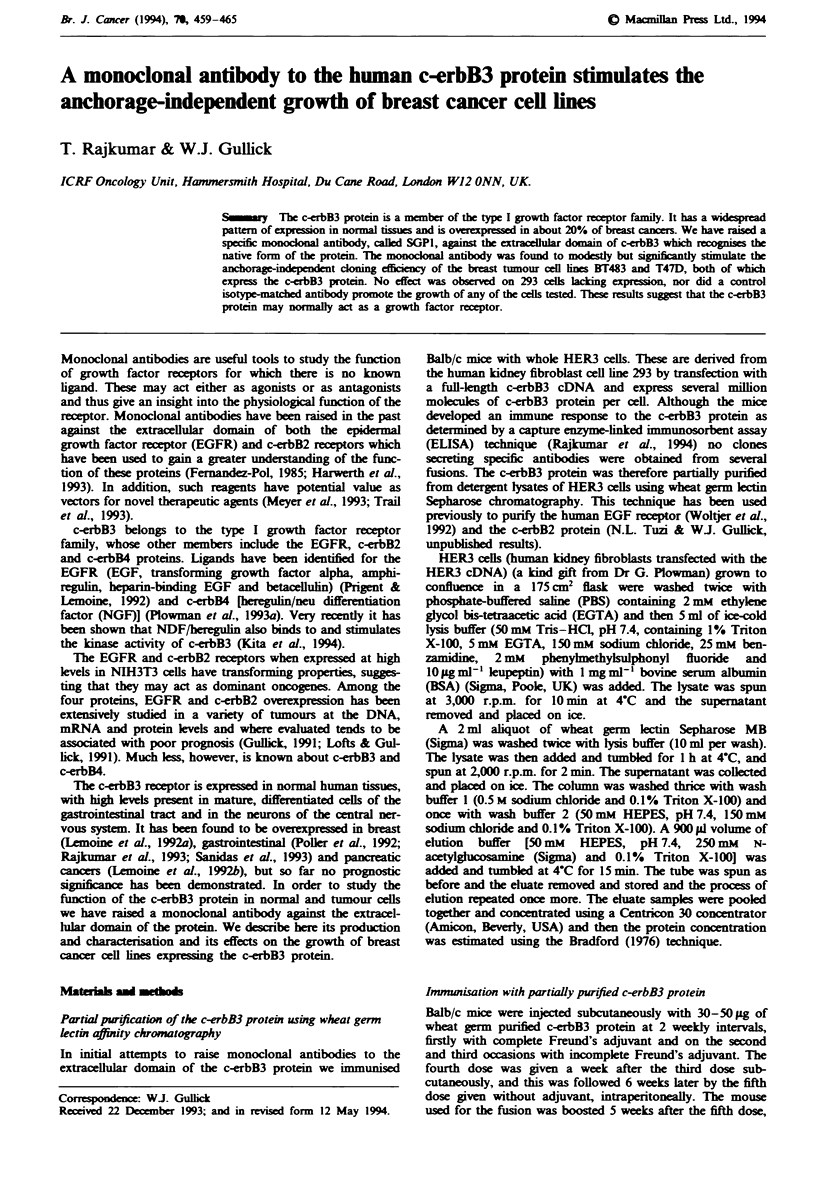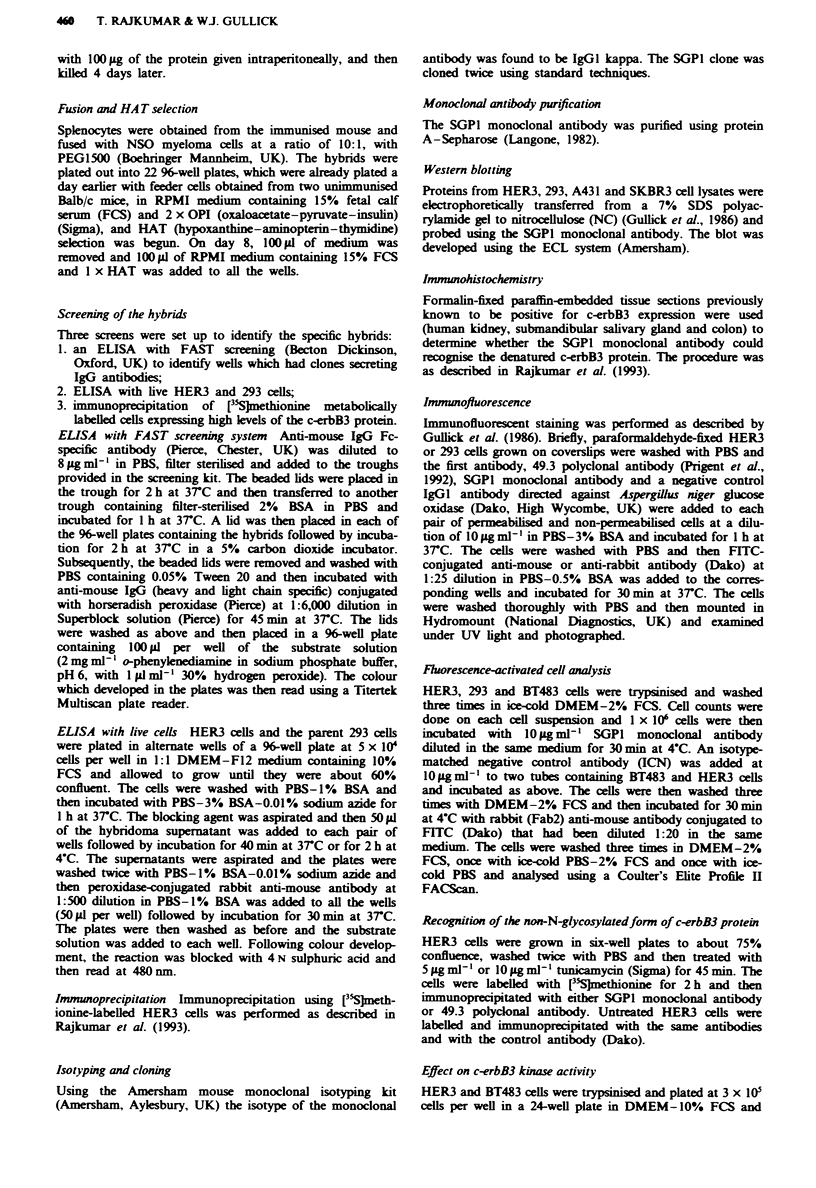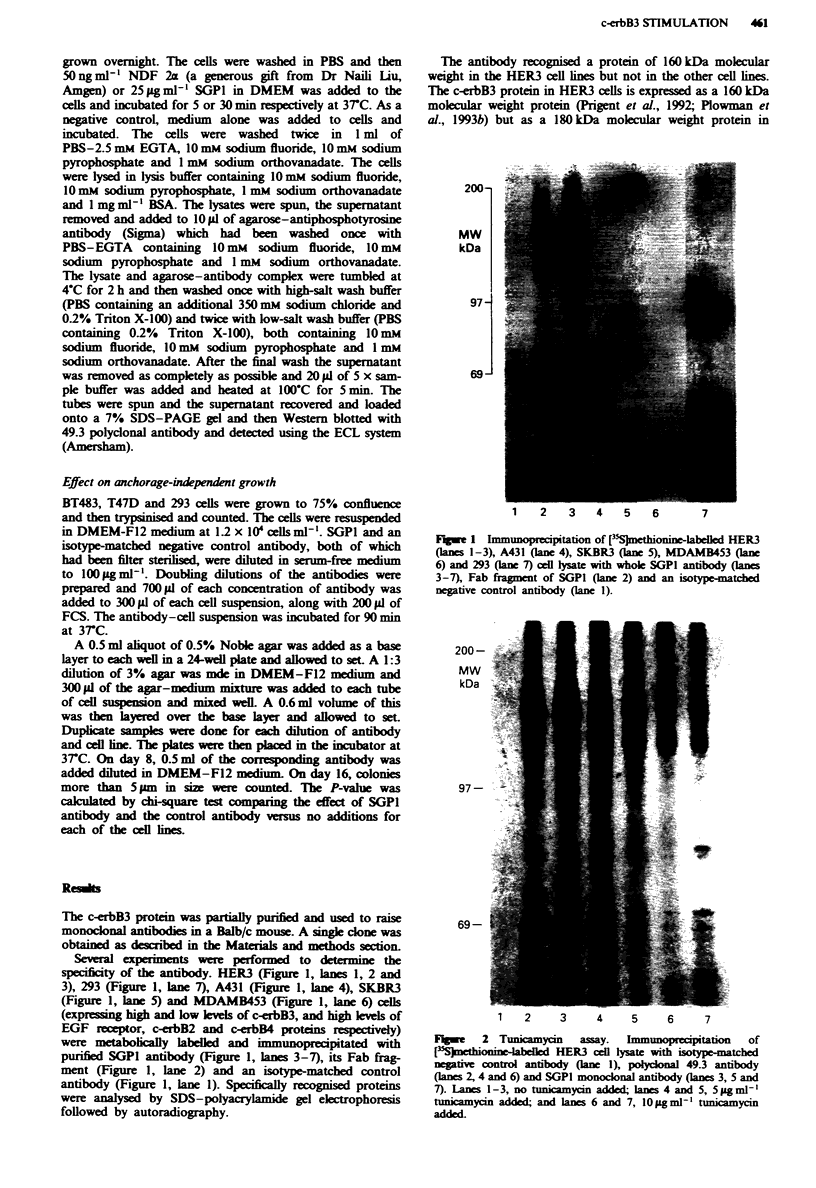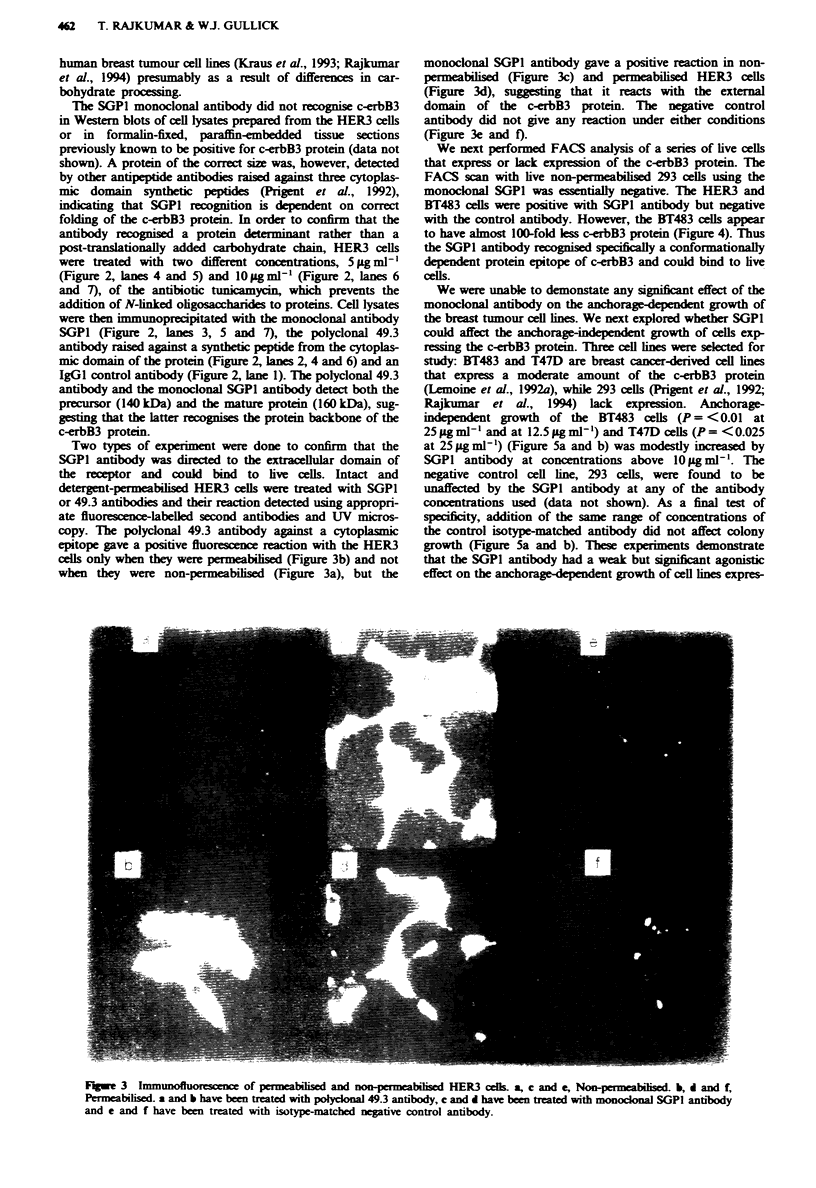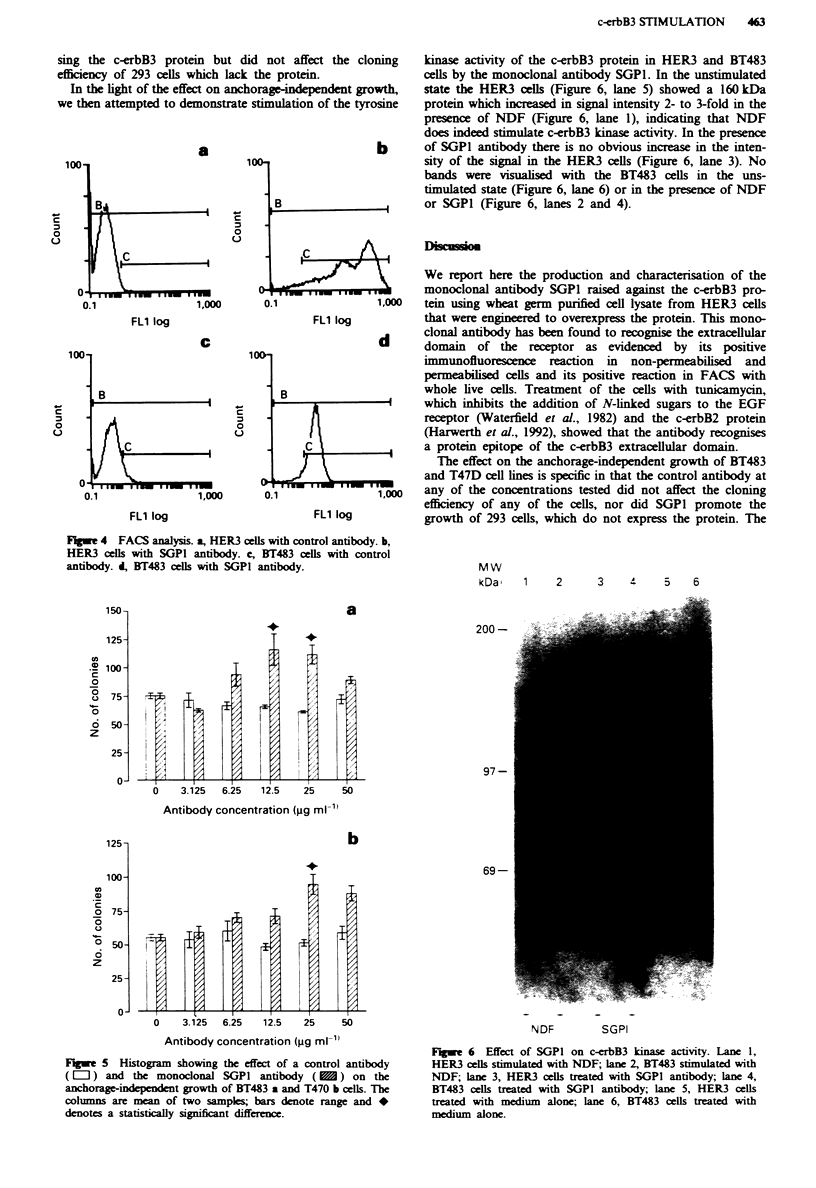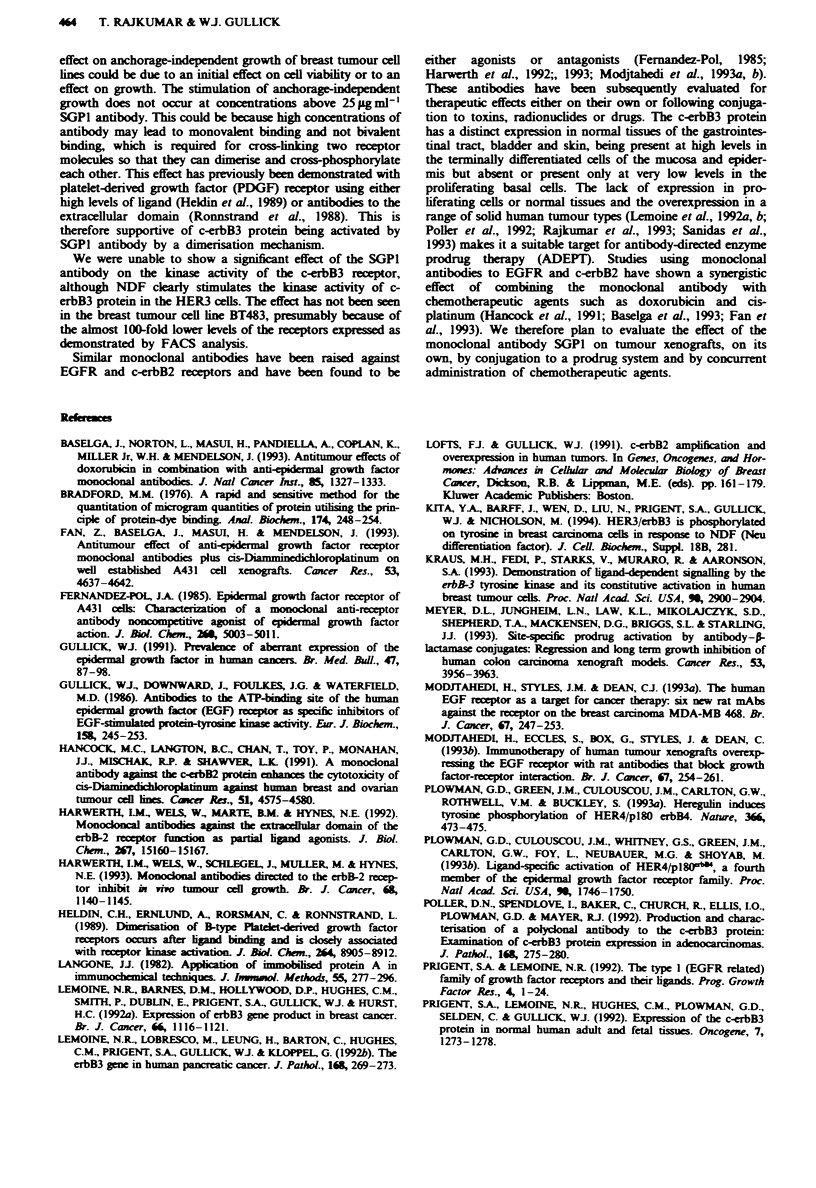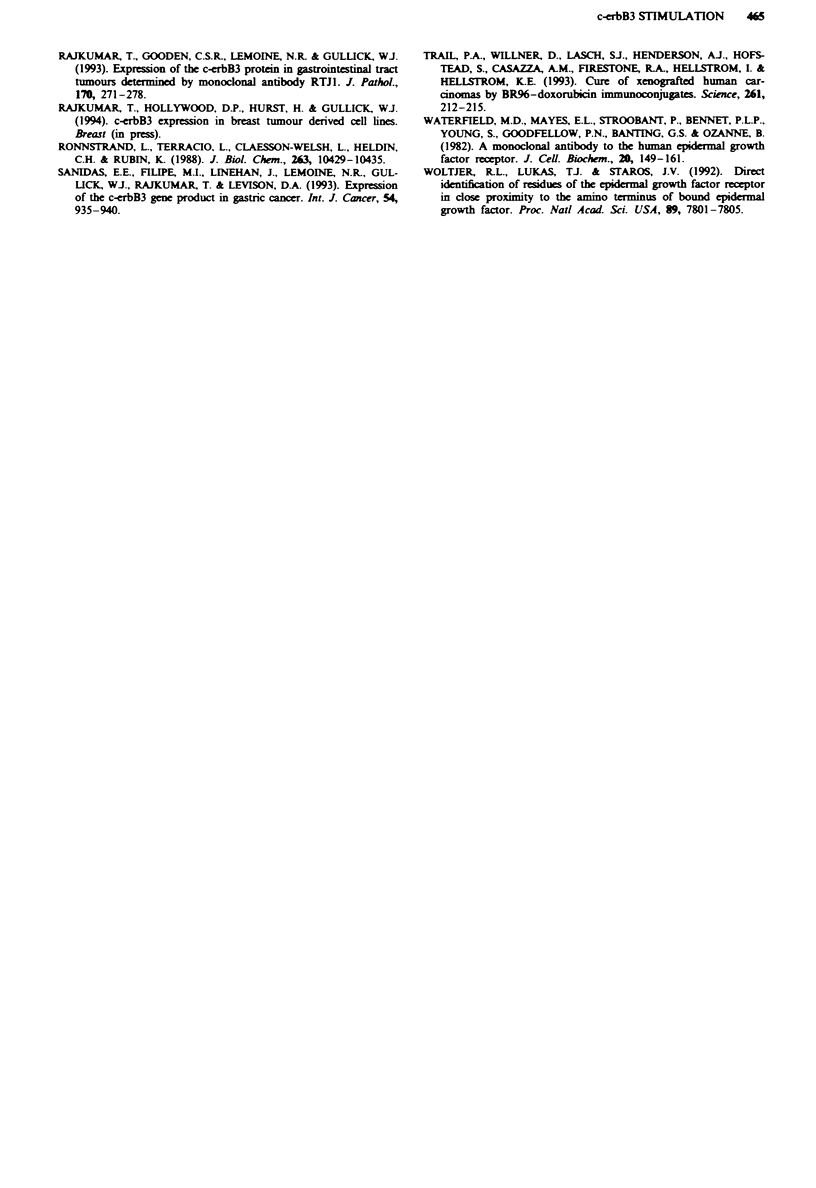# A monoclonal antibody to the human c-erbB3 protein stimulates the anchorage-independent growth of breast cancer cell lines.

**DOI:** 10.1038/bjc.1994.328

**Published:** 1994-09

**Authors:** T. Rajkumar, W. J. Gullick

**Affiliations:** ICRF Oncology Unit, Hammersmith Hospital, London UK.

## Abstract

**Images:**


Br. J. Cancer (1994), 70, 459-465 © Macmillan Press Ltd., 1994

### A monoclonal antibody to the human c-erbB3 protein stimulates the anchorage-independent growth of breast cancer cell lines

T. Rajkumar & W.J. Gullick

*ICRF Oncology Unit, Hammersmith Hospital, Du Cane Road, London W12 0NN, UK.*

**Summary** The c-erbB3 protein is a member of the type I growth factor receptor family. It has a widespread pattern of expression in normal tissues and is overexpressed in about 20% of breast cancers. We have raised a specific monoclonal antibody, called SGP1, against the extracellular domain of c-erbB3 which recognises the native form of the protein. The monoclonal antibody was found to modestly but significantly stimulate the anchorage-independent cloning efficiency of the breast tumour cell lines BT483 and T47D, both of which express the c-erbB3 protein. No effect was observed on 293 cells lacking expression, nor did a control isotype-matched antibody promote the growth of any of the cells tested. These results suggest that the c-erbB3 protein may normally act as a growth factor receptor.

Monoclonal antibodies are useful tools to study the function of growth factor receptors for which there is no known ligand. These may act either as agonists or as antagonists and thus give an insight into the physiological function of the receptor. Monoclonal antibodies have been raised in the past against the extracellular domain of both the epidermal growth factor receptor (EGFR) and c-erbB2 receptors which have been used to gain a greater understanding of the function of these proteins (Fernandez-Pol, 1985; Harwerth *et al.*, 1993). In addition, such reagents have potential value as vectors for novel therapeutic agents (Meyer *et al.*, 1993; Trail *et al.*, 1993).

c-erbB3 belongs to the type I growth factor receptor family, whose other members include the EGFR, c-erbB2 and c-erbB4 proteins. Ligands have been identified for the EGFR (EGF, transforming growth factor alpha, amphiregulin, heparin-binding EGF and betacellulin) (Prigent & Lemoine, 1992) and c-erbB4 [heregulin/neu differentiation factor (NGF)] (Plowman *et al.*, 1993*a*). Very recently it has been shown that NDF/heregulin also binds to and stimulates the kinase activity of c-erbB3 (Kita *et al.*, 1994).

The EGFR and c-erbB2 receptors when expressed at high levels in NIH3T3 cells have transforming properties, suggesting that they may act as dominant oncogenes. Among the four proteins, EGFR and c-erbB2 overexpression has been extensively studied in a variety of tumours at the DNA, mRNA and protein levels and where evaluated tends to be associated with poor prognosis (Gullick, 1991; Lofts & Gullick, 1991). Much less, however, is known about c-erbB3 and c-erbB4.

The c-erbB3 receptor is expressed in normal human tissues, with high levels present in mature, differentiated cells of the gastrointestinal tract and in the neurons of the central nervous system. It has been found to be overexpressed in breast (Lemoine *et al.*, 1992*a*), gastrointestinal (Poller *et al.*, 1992; Rajkumar *et al.*, 1993; Sanidas *et al.*, 1993) and pancreatic cancers (Lemoine *et al.*, 1992*b*), but so far no prognostic significance has been demonstrated. In order to study the function of the c-erbB3 protein in normal and tumour cells we have raised a monoclonal antibody against the extracellular domain of the protein. We describe here its production and characterisation and its effects on the growth of breast cancer cell lines expressing the c-erbB3 protein.

### Materials and methods

### *Partial purification of the c-erbB3 protein using wheat germ lectin affinity chromatography*

In initial attempts to raise monoclonal antibodies to the extracellular domain of the c-erbB3 protein we immunised Balb/c mice with whole HER3 cells. These are derived from the human kidney fibroblast cell line 293 by transfection with a full-length c-erbB3 cDNA and express several million molecules of c-erbB3 protein per cell. Although the mice developed an immune response to the c-erbB3 protein as determined by a capture enzyme-linked immunosorbent assay (ELISA) technique (Rajkumar *et al.*, 1994) no clones secreting specific antibodies were obtained from several fusions. The c-erbB3 protein was therefore partially purified from detergent lysates of HER3 cells using wheat germ lectin Sepharose chromatography. This technique has been used previously to purify the human EGF receptor (Woltjer *et al.*, 1992) and the c-erbB2 protein (N.L. Tuzi & W.J. Gullick, unpublished results).

HER3 cells (human kidney fibroblasts transfected with the HER3 cDNA) (a kind gift from Dr G. Plowman) grown to confluence in a 175 $cm^2$ flask were washed twice with phosphate-buffered saline (PBS) containing 2 mM ethylene glycol bis-tetraacetic acid (EGTA) and then 5 ml of ice-cold lysis buffer (50 mM Tris-HCl, pH 7.4, containing 1% Triton X-100, 5 mM EGTA, 150 mM sodium chloride, 25 mM benzamidine, 2 mM phenylmethylsulphonyl fluoride and 10 $\mu g\ ml^{-1}$ leupeptin) with 1 $mg\ ml^{-1}$ bovine serum albumin (BSA) (Sigma, Poole, UK) was added. The lysate was spun at 3,000 r.p.m. for 10 min at 4°C and the supernatant removed and placed on ice.

A 2 ml aliquot of wheat germ lectin Sepharose MB (Sigma) was washed twice with lysis buffer (10 ml per wash). The lysate was then added and tumbled for 1 h at 4°C, and spun at 2,000 r.p.m. for 2 min. The supernatant was collected and placed on ice. The column was washed thrice with wash buffer 1 (0.5 M sodium chloride and 0.1% Triton X-100) and once with wash buffer 2 (50 mM HEPES, pH 7.4, 150 mM sodium chloride and 0.1% Triton X-100). A 900 µl volume of elution buffer [50 mM HEPES, pH 7.4, 250 mM N-acetylglucosamine (Sigma) and 0.1% Triton X-100] was added and tumbled at 4°C for 15 min. The tube was spun as before and the eluate removed and stored and the process of elution repeated once more. The eluate samples were pooled together and concentrated using a Centricon 30 concentrator (Amicon, Beverly, USA) and then the protein concentration was estimated using the Bradford (1976) technique.

### *Immunisation with partially purified c-erbB3 protein*

Balb/c mice were injected subcutaneously with 30-50 µg of wheat germ purified c-erbB3 protein at 2 weekly intervals, firstly with complete Freund's adjuvant and on the second and third occasions with incomplete Freund's adjuvant. The fourth dose was given a week after the third dose subcutaneously, and this was followed 6 weeks later by the fifth dose given without adjuvant, intraperitoneally. The mouse used for the fusion was boosted 5 weeks after the fifth dose,

Correspondence: W.J. Gullick
Received 22 December 1993; and in revised form 12 May 1994.

with 100 μg of the protein given intraperitoneally, and then killed 4 days later.

*Fusion and HAT selection*

Splenocytes were obtained from the immunised mouse and fused with NSO myeloma cells at a ratio of 10:1, with PEG1500 (Boehringer Mannheim, UK). The hybrids were plated out into 22 96-well plates, which were already plated a day earlier with feeder cells obtained from two unimmunised Balb/c mice, in RPMI medium containing 15% fetal calf serum (FCS) and 2 × OPI (oxaloacetate–pyruvate–insulin) (Sigma), and HAT (hypoxanthine–aminopterin–thymidine) selection was begun. On day 8, 100 μl of medium was removed and 100 μl of RPMI medium containing 15% FCS and 1 × HAT was added to all the wells.

*Screening of the hybrids*

Three screens were set up to identify the specific hybrids:
1. an ELISA with FAST screening (Becton Dickinson, Oxford, UK) to identify wells which had clones secreting IgG antibodies;
2. ELISA with live HER3 and 293 cells;
3. immunoprecipitation of [$^{35}$S]methionine metabolically labelled cells expressing high levels of the c-erbB3 protein.

*ELISA with FAST screening system* Anti-mouse IgG Fc-specific antibody (Pierce, Chester, UK) was diluted to 8 μg ml$^{-1}$ in PBS, filter sterilised and added to the troughs provided in the screening kit. The beaded lids were placed in the trough for 2 h at 37°C and then transferred to another trough containing filter-sterilised 2% BSA in PBS and incubated for 1 h at 37°C. A lid was then placed in each of the 96-well plates containing the hybrids followed by incubation for 2 h at 37°C in a 5% carbon dioxide incubator. Subsequently, the beaded lids were removed and washed with PBS containing 0.05% Tween 20 and then incubated with anti-mouse IgG (heavy and light chain specific) conjugated with horseradish peroxidase (Pierce) at 1:6,000 dilution in Superblock solution (Pierce) for 45 min at 37°C. The lids were washed as above and then placed in a 96-well plate containing 100 μl per well of the substrate solution (2 mg ml$^{-1}$ *o*-phenylenediamine in sodium phosphate buffer, pH 6, with 1 μl ml$^{-1}$ 30% hydrogen peroxide). The colour which developed in the plates was then read using a Titertek Multiscan plate reader.

*ELISA with live cells* HER3 cells and the parent 293 cells were plated in alternate wells of a 96-well plate at 5 × 10$^4$ cells per well in 1:1 DMEM–F12 medium containing 10% FCS and allowed to grow until they were about 60% confluent. The cells were washed with PBS–1% BSA and then incubated with PBS–3% BSA–0.01% sodium azide for 1 h at 37°C. The blocking agent was aspirated and then 50 μl of the hybridoma supernatant was added to each pair of wells followed by incubation for 40 min at 37°C or for 2 h at 4°C. The supernatants were aspirated and the plates were washed twice with PBS–1% BSA–0.01% sodium azide and then peroxidase-conjugated rabbit anti-mouse antibody at 1:500 dilution in PBS–1% BSA was added to all the wells (50 μl per well) followed by incubation for 30 min at 37°C. The plates were then washed as before and the substrate solution was added to each well. Following colour development, the reaction was blocked with 4 N sulphuric acid and then read at 480 nm.

*Immunoprecipitation* Immunoprecipitation using [$^{35}$S]methionine-labelled HER3 cells was performed as described in Rajkumar *et al.* (1993).

*Isotyping and cloning*

Using the Amersham mouse monoclonal isotyping kit (Amersham, Aylesbury, UK) the isotype of the monoclonal antibody was found to be IgG1 kappa. The SGP1 clone was cloned twice using standard techniques.

*Monoclonal antibody purification*

The SGP1 monoclonal antibody was purified using protein A–Sepharose (Langone, 1982).

*Western blotting*

Proteins from HER3, 293, A431 and SKBR3 cell lysates were electrophoretically transferred from a 7% SDS polyacrylamide gel to nitrocellulose (NC) (Gullick *et al.*, 1986) and probed using the SGP1 monoclonal antibody. The blot was developed using the ECL system (Amersham).

*Immunohistochemistry*

Formalin-fixed paraffin-embedded tissue sections previously known to be positive for c-erbB3 expression were used (human kidney, submandibular salivary gland and colon) to determine whether the SGP1 monoclonal antibody could recognise the denatured c-erbB3 protein. The procedure was as described in Rajkumar *et al.* (1993).

*Immunofluorescence*

Immunofluorescent staining was performed as described by Gullick *et al.* (1986). Briefly, paraformaldehyde-fixed HER3 or 293 cells grown on coverslips were washed with PBS and the first antibody, 49.3 polyclonal antibody (Prigent *et al.*, 1992), SGP1 monoclonal antibody and a negative control IgG1 antibody directed against *Aspergillus niger* glucose oxidase (Dako, High Wycombe, UK) were added to each pair of permeabilised and non-permeabilised cells at a dilution of 10 μg ml$^{-1}$ in PBS–3% BSA and incubated for 1 h at 37°C. The cells were washed with PBS and then FITC-conjugated anti-mouse or anti-rabbit antibody (Dako) at 1:25 dilution in PBS–0.5% BSA was added to the corresponding wells and incubated for 30 min at 37°C. The cells were washed thoroughly with PBS and then mounted in Hydromount (National Diagnostics, UK) and examined under UV light and photographed.

*Fluorescence-activated cell analysis*

HER3, 293 and BT483 cells were trypsinised and washed three times in ice-cold DMEM–2% FCS. Cell counts were done on each cell suspension and 1 × 10$^6$ cells were then incubated with 10 μg ml$^{-1}$ SGP1 monoclonal antibody diluted in the same medium for 30 min at 4°C. An isotype-matched negative control antibody (ICN) was added at 10 μg ml$^{-1}$ to two tubes containing BT483 and HER3 cells and incubated as above. The cells were then washed three times with DMEM–2% FCS and then incubated for 30 min at 4°C with rabbit (Fab2) anti-mouse antibody conjugated to FITC (Dako) that had been diluted 1:20 in the same medium. The cells were washed three times in DMEM–2% FCS, once with ice-cold PBS–2% FCS and once with ice-cold PBS and analysed using a Coulter's Elite Profile II FACScan.

*Recognition of the non-*N*-glycosylated form of c-erbB3 protein*

HER3 cells were grown in six-well plates to about 75% confluence, washed twice with PBS and then treated with 5 μg ml$^{-1}$ or 10 μg ml$^{-1}$ tunicamycin (Sigma) for 45 min. The cells were labelled with [$^{35}$S]methionine for 2 h and then immunoprecipitated with either SGP1 monoclonal antibody or 49.3 polyclonal antibody. Untreated HER3 cells were labelled and immunoprecipitated with the same antibodies and with the control antibody (Dako).

*Effect on c-erbB3 kinase activity*

HER3 and BT483 cells were trypsinised and plated at 3 × 10$^5$ cells per well in a 24-well plate in DMEM–10% FCS and grown overnight. The cells were washed in PBS and then 50 ng $ml^{-1}$ NDF 2α (a generous gift from Dr Naili Liu, Amgen) or 25 μg $ml^{-1}$ SGP1 in DMEM was added to the cells and incubated for 5 or 30 min respectively at 37°C. As a negative control, medium alone was added to cells and incubated. The cells were washed twice in 1 ml of PBS-2.5 mM EGTA, 10 mM sodium fluoride, 10 mM sodium pyrophosphate and 1 mM sodium orthovanadate. The cells were lysed in lysis buffer containing 10 mM sodium fluoride, 10 mM sodium pyrophosphate, 1 mM sodium orthovanadate and 1 mg $ml^{-1}$ BSA. The lysates were spun, the supernatant removed and added to 10 μl of agarose-antiphosphotyrosine antibody (Sigma) which had been washed once with PBS-EGTA containing 10 mM sodium fluoride, 10 mM sodium pyrophosphate and 1 mM sodium orthovanadate. The lysate and agarose-antibody complex were tumbled at 4°C for 2 h and then washed once with high-salt wash buffer (PBS containing an additional 350 mM sodium chloride and 0.2% Triton X-100) and twice with low-salt wash buffer (PBS containing 0.2% Triton X-100), both containing 10 mM sodium fluoride, 10 mM sodium pyrophosphate and 1 mM sodium orthovanadate. After the final wash the supernatant was removed as completely as possible and 20 μl of 5 × sample buffer was added and heated at 100°C for 5 min. The tubes were spun and the supernatant recovered and loaded onto a 7% SDS-PAGE gel and then Western blotted with 49.3 polyclonal antibody and detected using the ECL system (Amersham).

*Effect on anchorage-independent growth*

BT483, T47D and 293 cells were grown to 75% confluence and then trypsinised and counted. The cells were resuspended in DMEM-F12 medium at 1.2 × $10^4$ cells $ml^{-1}$. SGP1 and an isotype-matched negative control antibody, both of which had been filter sterilised, were diluted in serum-free medium to 100 μg $ml^{-1}$. Doubling dilutions of the antibodies were prepared and 700 μl of each concentration of antibody was added to 300 μl of each cell suspension, along with 200 μl of FCS. The antibody-cell suspension was incubated for 90 min at 37°C.

A 0.5 ml aliquot of 0.5% Noble agar was added as a base layer to each well in a 24-well plate and allowed to set. A 1:3 dilution of 3% agar was mde in DMEM-F12 medium and 300 μl of the agar-medium mixture was added to each tube of cell suspension and mixed well. A 0.6 ml volume of this was then layered over the base layer and allowed to set. Duplicate samples were done for each dilution of antibody and cell line. The plates were then placed in the incubator at 37°C. On day 8, 0.5 ml of the corresponding antibody was added diluted in DMEM-F12 medium. On day 16, colonies more than 5 μm in size were counted. The *P*-value was calculated by chi-square test comparing the effect of SGP1 antibody and the control antibody versus no additions for each of the cell lines.

### Results

The c-erbB3 protein was partially purified and used to raise monoclonal antibodies in a Balb/c mouse. A single clone was obtained as described in the Materials and methods section.

Several experiments were performed to determine the specificity of the antibody. HER3 (Figure 1, lanes 1, 2 and 3), 293 (Figure 1, lane 7), A431 (Figure 1, lane 4), SKBR3 (Figure 1, lane 5) and MDAMB453 (Figure 1, lane 6) cells (expressing high and low levels of c-erbB3, and high levels of EGF receptor, c-erbB2 and c-erbB4 proteins respectively) were metabolically labelled and immunoprecipitated with purified SGP1 antibody (Figure 1, lanes 3-7), its Fab fragment (Figure 1, lane 2) and an isotype-matched control antibody (Figure 1, lane 1). Specifically recognised proteins were analysed by SDS-polyacrylamide gel electrophoresis followed by autoradiography.

The antibody recognised a protein of 160 kDa molecular weight in the HER3 cell lines but not in the other cell lines. The c-erbB3 protein in HER3 cells is expressed as a 160 kDa molecular weight protein (Prigent *et al.*, 1992; Plowman *et al.*, 1993*b*) but as a 180 kDa molecular weight protein in

**Figure 1** Immunoprecipitation of [$^{35}$S]methionine-labelled HER3 (lanes 1-3), A431 (lane 4), SKBR3 (lane 5), MDAMB453 (lane 6) and 293 (lane 7) cell lysate with whole SGP1 antibody (lanes 3-7), Fab fragment of SGP1 (lane 2) and an isotype-matched negative control antibody (lane 1).

**Figure 2** Tunicamycin assay. Immunoprecipitation of [$^{35}$S]methionine-labelled HER3 cell lysate with isotype-matched negative control antibody (lane 1), polyclonal 49.3 antibody (lanes 2, 4 and 6) and SGP1 monoclonal antibody (lanes 3, 5 and 7). Lanes 1-3, no tunicamycin added; lanes 4 and 5, 5 μg $ml^{-1}$ tunicamycin added; and lanes 6 and 7, 10 μg $ml^{-1}$ tunicamycin added.

human breast tumour cell lines (Kraus *et al.*, 1993; Rajkumar *et al.*, 1994) presumably as a result of differences in carbohydrate processing.

The SGP1 monoclonal antibody did not recognise c-erbB3 in Western blots of cell lysates prepared from the HER3 cells or in formalin-fixed, paraffin-embedded tissue sections previously known to be positive for c-erbB3 protein (data not shown). A protein of the correct size was, however, detected by other antipeptide antibodies raised against three cytoplasmic domain synthetic peptides (Prigent *et al.*, 1992), indicating that SGP1 recognition is dependent on correct folding of the c-erbB3 protein. In order to confirm that the antibody recognised a protein determinant rather than a post-translationally added carbohydrate chain, HER3 cells were treated with two different concentrations, 5 $\mu g\ ml^{-1}$ (Figure 2, lanes 4 and 5) and 10 $\mu g\ ml^{-1}$ (Figure 2, lanes 6 and 7), of the antibiotic tunicamycin, which prevents the addition of *N*-linked oligosaccharides to proteins. Cell lysates were then immunoprecipitated with the monoclonal antibody SGP1 (Figure 2, lanes 3, 5 and 7), the polyclonal 49.3 antibody raised against a synthetic peptide from the cytoplasmic domain of the protein (Figure 2, lanes 2, 4 and 6) and an IgG1 control antibody (Figure 2, lane 1). The polyclonal 49.3 antibody and the monoclonal SGP1 antibody detect both the precursor (140 kDa) and the mature protein (160 kDa), suggesting that the latter recognises the protein backbone of the c-erbB3 protein.

Two types of experiment were done to confirm that the SGP1 antibody was directed to the extracellular domain of the receptor and could bind to live cells. Intact and detergent-permeabilised HER3 cells were treated with SGP1 or 49.3 antibodies and their reaction detected using appropriate fluorescence-labelled second antibodies and UV microscopy. The polyclonal 49.3 antibody against a cytoplasmic epitope gave a positive fluorescence reaction with the HER3 cells only when they were permeabilised (Figure 3b) and not when they were non-permeabilised (Figure 3a), but the monoclonal SGP1 antibody gave a positive reaction in non-permeabilised (Figure 3c) and permeabilised HER3 cells (Figure 3d), suggesting that it reacts with the external domain of the c-erbB3 protein. The negative control antibody did not give any reaction under either conditions (Figure 3e and f).

We next performed FACS analysis of a series of live cells that express or lack expression of the c-erbB3 protein. The FACS scan with live non-permeabilised 293 cells using the monoclonal SGP1 was essentially negative. The HER3 and BT483 cells were positive with SGP1 antibody but negative with the control antibody. However, the BT483 cells appear to have almost 100-fold less c-erbB3 protein (Figure 4). Thus the SGP1 antibody recognised specifically a conformationally dependent protein epitope of c-erbB3 and could bind to live cells.

We were unable to demonstate any significant effect of the monoclonal antibody on the anchorage-dependent growth of the breast tumour cell lines. We next explored whether SGP1 could affect the anchorage-independent growth of cells expressing the c-erbB3 protein. Three cell lines were selected for study: BT483 and T47D are breast cancer-derived cell lines that express a moderate amount of the c-erbB3 protein (Lemoine *et al.*, 1992*a*), while 293 cells (Prigent *et al.*, 1992; Rajkumar *et al.*, 1994) lack expression. Anchorage-independent growth of the BT483 cells ($P = <0.01$ at 25 $\mu g\ ml^{-1}$ and at 12.5 $\mu g\ ml^{-1}$) and T47D cells ($P = <0.025$ at 25 $\mu g\ ml^{-1}$) (Figure 5a and b) was modestly increased by SGP1 antibody at concentrations above 10 $\mu g\ ml^{-1}$. The negative control cell line, 293 cells, were found to be unaffected by the SGP1 antibody at any of the antibody concentrations used (data not shown). As a final test of specificity, addition of the same range of concentrations of the control isotype-matched antibody did not affect colony growth (Figure 5a and b). These experiments demonstrate that the SGP1 antibody had a weak but significant agonistic effect on the anchorage-dependent growth of cell lines expres-

**Figure 3** Immunofluorescence of permeabilised and non-permeabilised HER3 cells. **a**, **c** and **e**, Non-permeabilised. **b**, **d** and **f**, Permeabilised. **a** and **b** have been treated with polyclonal 49.3 antibody, **c** and **d** have been treated with monoclonal SGP1 antibody and **e** and **f** have been treated with isotype-matched negative control antibody.

sing the c-erbB3 protein but did not affect the cloning efficiency of 293 cells which lack the protein.

In the light of the effect on anchorage-independent growth, we then attempted to demonstrate stimulation of the tyrosine kinase activity of the c-erbB3 protein in HER3 and BT483 cells by the monoclonal antibody SGP1. In the unstimulated state the HER3 cells (Figure 6, lane 5) showed a 160 kDa protein which increased in signal intensity 2- to 3-fold in the presence of NDF (Figure 6, lane 1), indicating that NDF does indeed stimulate c-erbB3 kinase activity. In the presence of SGP1 antibody there is no obvious increase in the intensity of the signal in the HER3 cells (Figure 6, lane 3). No bands were visualised with the BT483 cells in the unstimulated state (Figure 6, lane 6) or in the presence of NDF or SGP1 (Figure 6, lanes 2 and 4).

**Figure 4** FACS analysis. **a**, HER3 cells with control antibody. **b**, HER3 cells with SGP1 antibody. **c**, BT483 cells with control antibody. **d**, BT483 cells with SGP1 antibody.

**Figure 5** Histogram showing the effect of a control antibody (□) and the monoclonal SGP1 antibody (▨) on the anchorage-independent growth of BT483 **a** and T470 **b** cells. The columns are mean of two samples; bars denote range and ◆ denotes a statistically significant difference.

### Discussion

We report here the production and characterisation of the monoclonal antibody SGP1 raised against the c-erbB3 protein using wheat germ purified cell lysate from HER3 cells that were engineered to overexpress the protein. This monoclonal antibody has been found to recognise the extracellular domain of the receptor as evidenced by its positive immunofluorescence reaction in non-permeabilised and permeabilised cells and its positive reaction in FACS with whole live cells. Treatment of the cells with tunicamycin, which inhibits the addition of *N*-linked sugars to the EGF receptor (Waterfield *et al.*, 1982) and the c-erbB2 protein (Harwerth *et al.*, 1992), showed that the antibody recognises a protein epitope of the c-erbB3 extracellular domain.

The effect on the anchorage-independent growth of BT483 and T47D cell lines is specific in that the control antibody at any of the concentrations tested did not affect the cloning efficiency of any of the cells, nor did SGP1 promote the growth of 293 cells, which do not express the protein. The

**Figure 6** Effect of SGP1 on c-erbB3 kinase activity. Lane 1, HER3 cells stimulated with NDF; lane 2, BT483 stimulated with NDF; lane 3, HER3 cells treated with SGP1 antibody; lane 4, BT483 cells treated with SGP1 antibody; lane 5, HER3 cells treated with medium alone; lane 6, BT483 cells treated with medium alone.

effect on anchorage-independent growth of breast tumour cell lines could be due to an initial effect on cell viability or to an effect on growth. The stimulation of anchorage-independent growth does not occur at concentrations above 25 $\mu g\ ml^{-1}$ SGP1 antibody. This could be because high concentrations of antibody may lead to monovalent binding and not bivalent binding, which is required for cross-linking two receptor molecules so that they can dimerise and cross-phosphorylate each other. This effect has previously been demonstrated with platelet-derived growth factor (PDGF) receptor using either high levels of ligand (Heldin *et al.*, 1989) or antibodies to the extracellular domain (Ronnstrand *et al.*, 1988). This is therefore supportive of c-erbB3 protein being activated by SGP1 antibody by a dimerisation mechanism.

We were unable to show a significant effect of the SGP1 antibody on the kinase activity of the c-erbB3 receptor, although NDF clearly stimulates the kinase activity of c-erbB3 protein in the HER3 cells. The effect has not been seen in the breast tumour cell line BT483, presumably because of the almost 100-fold lower levels of the receptors expressed as demonstrated by FACS analysis.

Similar monoclonal antibodies have been raised against EGFR and c-erbB2 receptors and have been found to be either agonists or antagonists (Fernandez-Pol, 1985; Harwerth *et al.*, 1992;, 1993; Modjtahedi *et al.*, 1993*a*, *b*). These antibodies have been subsequently evaluated for therapeutic effects either on their own or following conjugation to toxins, radionuclides or drugs. The c-erbB3 protein has a distinct expression in normal tissues of the gastrointestinal tract, bladder and skin, being present at high levels in the terminally differentiated cells of the mucosa and epidermis but absent or present only at very low levels in the proliferating basal cells. The lack of expression in proliferating cells or normal tissues and the overexpression in a range of solid human tumour types (Lemoine *et al.*, 1992*a*, *b*; Poller *et al.*, 1992; Rajkumar *et al.*, 1993; Sanidas *et al.*, 1993) makes it a suitable target for antibody-directed enzyme prodrug therapy (ADEPT). Studies using monoclonal antibodies to EGFR and c-erbB2 have shown a synergistic effect of combining the monoclonal antibody with chemotherapeutic agents such as doxorubicin and cisplatinum (Hancock *et al.*, 1991; Baselga *et al.*, 1993; Fan *et al.*, 1993). We therefore plan to evaluate the effect of the monoclonal antibody SGP1 on tumour xenografts, on its own, by conjugation to a prodrug system and by concurrent administration of chemotherapeutic agents.

### References

BASELGA, J., NORTON, L., MASUI, H., PANDIELLA, A., COPLAN, K., MILLER Jr, W.H. & MENDELSON, J. (1993). Antitumour effects of doxorubicin in combination with anti-epidermal growth factor monoclonal antibodies. *J. Natl Cancer Inst.*, **85**, 1327–1333.

BRADFORD, M.M. (1976). A rapid and sensitive method for the quantitation of microgram quantities of protein utilising the principle of protein-dye binding. *Anal. Biochem.*, **174**, 248–254.

FAN, Z., BASELGA, J., MASUI, H. & MENDELSON, J. (1993). Antitumour effect of anti-epidermal growth factor receptor monoclonal antibodies plus cis-Diamminedichloroplatinum on well established A431 cell xenografts. *Cancer Res.*, **53**, 4637–4642.

FERNANDEZ-POL, J.A. (1985). Epidermal growth factor receptor of A431 cells: Characterization of a monoclonal anti-receptor antibody noncompetitive agonist of epidermal growth factor action. *J. Biol. Chem.*, **260**, 5003–5011.

GULLICK, W.J. (1991). Prevalence of aberrant expression of the epidermal growth factor in human cancers. *Br. Med. Bull.*, **47**, 87–98.

GULLICK, W.J., DOWNWARD, J., FOULKES, J.G. & WATERFIELD, M.D. (1986). Antibodies to the ATP-binding site of the human epidermal growth factor (EGF) receptor as specific inhibitors of EGF-stimulated protein-tyrosine kinase activity. *Eur. J. Biochem.*, **158**, 245–253.

HANCOCK, M.C., LANGTON, B.C., CHAN, T., TOY, P., MONAHAN, J.J., MISCHAK, R.P. & SHAWVER, L.K. (1991). A monoclonal antibody against the c-erbB2 protein enhances the cytotoxicity of cis-Diaminedichloroplatinum against human breast and ovarian tumour cell lines. *Cancer Res.*, **51**, 4575–4580.

HARWERTH, I.M., WELS, W., MARTE, B.M. & HYNES, N.E. (1992). Monoclonal antibodies against the extracellular domain of the erbB-2 receptor function as partial ligand agonists. *J. Biol. Chem.*, **267**, 15160–15167.

HARWERTH, I.M., WELS, W., SCHLEGEL, J., MULLER, M. & HYNES, N.E. (1993). Monoclonal antibodies directed to the erbB-2 receptor inhibit *in vivo* tumour cell growth. *Br. J. Cancer*, **68**, 1140–1145.

HELDIN, C.H., ERNLUND, A., RORSMAN, C. & RONNSTRAND, L. (1989). Dimerisation of B-type Platelet-derived growth factor receptors occurs after ligand binding and is closely associated with receptor kinase activation. *J. Biol. Chem.*, **264**, 8905–8912.

LANGONE, J.J. (1982). Application of immobilised protein A in immunochemical techniques. *J. Immunol. Methods*, **55**, 277–296.

LEMOINE, N.R., BARNES, D.M., HOLLYWOOD, D.P., HUGHES, C.M., SMITH, P., DUBLIN, E., PRIGENT, S.A., GULLICK, W.J. & HURST, H.C. (1992*a*). Expression of erbB3 gene product in breast cancer. *Br. J. Cancer*, **66**, 1116–1121.

LEMOINE, N.R., LOBRESCO, M., LEUNG, H., BARTON, C., HUGHES, C.M., PRIGENT, S.A., GULLICK, W.J. & KLOPPEL, G. (1992*b*). The erbB3 gene in human pancreatic cancer. *J. Pathol.*, **168**, 269–273.

LOFTS, F.J. & GULLICK, W.J. (1991). c-erbB2 amplification and overexpression in human tumors. In *Genes, Oncogenes, and Hormones: Advances in Cellular and Molecular Biology of Breast Cancer*, Dickson, R.B. & Lippman, M.E. (eds). pp. 161–179. Kluwer Academic Publishers: Boston.

KITA, Y.A., BARFF, J., WEN, D., LIU, N., PRIGENT, S.A., GULLICK, W.J. & NICHOLSON, M. (1994). HER3/erbB3 is phosphorylated on tyrosine in breast carcinoma cells in response to NDF (Neu differentiation factor). *J. Cell. Biochem.*, Suppl. 18B, 281.

KRAUS, M.H., FEDI, P., STARKS, V., MURARO, R. & AARONSON, S.A. (1993). Demonstration of ligand-dependent signalling by the *erbB-3* tyrosine kinase and its constitutive activation in human breast tumour cells. *Proc. Natl Acad. Sci. USA*, **90**, 2900–2904.

MEYER, D.L., JUNGHEIM, L.N., LAW, K.L., MIKOLAJCZYK, S.D., SHEPHERD, T.A., MACKENSEN, D.G., BRIGGS, S.L. & STARLING, J.J. (1993). Site-specific prodrug activation by antibody-β-lactamase conjugates: Regression and long term growth inhibition of human colon carcinoma xenograft models. *Cancer Res.*, **53**, 3956–3963.

MODJTAHEDI, H., STYLES, J.M. & DEAN, C.J. (1993*a*). The human EGF receptor as a target for cancer therapy: six new rat mAbs against the receptor on the breast carcinoma MDA-MB 468. *Br. J. Cancer*, **67**, 247–253.

MODJTAHEDI, H., ECCLES, S., BOX, G., STYLES, J. & DEAN, C. (1993*b*). Immunotherapy of human tumour xenografts overexpressing the EGF receptor with rat antibodies that block growth factor-receptor interaction. *Br. J. Cancer*, **67**, 254–261.

PLOWMAN, G.D., GREEN, J.M., CULOUSCOU, J.M., CARLTON, G.W., ROTHWELL, V.M. & BUCKLEY, S. (1993*a*). Heregulin induces tyrosine phosphorylation of HER4/p180 erbB4. *Nature*, **366**, 473–475.

PLOWMAN, G.D., CULOUSCOU, J.M., WHITNEY, G.S., GREEN, J.M., CARLTON, G.W., FOY, L., NEUBAUER, M.G. & SHOYAB, M. (1993*b*). Ligand-specific activation of HER4/p180$^{erbB4}$, a fourth member of the epidermal growth factor receptor family. *Proc. Natl Acad. Sci. USA*, **90**, 1746–1750.

POLLER, D.N., SPENDLOVE, I., BAKER, C., CHURCH, R., ELLIS, I.O., PLOWMAN, G.D. & MAYER, R.J. (1992). Production and characterisation of a polyclonal antibody to the c-erbB3 protein: Examination of c-erbB3 protein expression in adenocarcinomas. *J. Pathol.*, **168**, 275–280.

PRIGENT, S.A. & LEMOINE, N.R. (1992). The type 1 (EGFR related) family of growth factor receptors and their ligands. *Prog. Growth Factor Res.*, **4**, 1–24.

PRIGENT, S.A., LEMOINE, N.R., HUGHES, C.M., PLOWMAN, G.D., SELDEN, C. & GULLICK, W.J. (1992). Expression of the c-erbB3 protein in normal human adult and fetal tissues. *Oncogene*, **7**, 1273–1278.

RAJKUMAR, T., GOODEN, C.S.R., LEMOINE, N.R. & GULLICK, W.J. (1993). Expression of the c-erbB3 protein in gastrointestinal tract tumours determined by monoclonal antibody RTJ1. *J. Pathol.*, **170**, 271-278.

RAJKUMAR, T., HOLLYWOOD, D.P., HURST, H. & GULLICK, W.J. (1994). c-erbB3 expression in breast tumour derived cell lines. *Breast* (in press).

RONNSTRAND, L., TERRACIO, L., CLAESSON-WELSH, L., HELDIN, C.H. & RUBIN, K. (1988). *J. Biol. Chem.*, **263**, 10429-10435.

SANIDAS, E.E., FILIPE, M.I., LINEHAN, J., LEMOINE, N.R., GULLICK, W.J., RAJKUMAR, T. & LEVISON, D.A. (1993). Expression of the c-erbB3 gene product in gastric cancer. *Int. J. Cancer*, **54**, 935-940.

TRAIL, P.A., WILLNER, D., LASCH, S.J., HENDERSON, A.J., HOFSTEAD, S., CASAZZA, A.M., FIRESTONE, R.A., HELLSTROM, I. & HELLSTROM, K.E. (1993). Cure of xenografted human carcinomas by BR96-doxorubicin immunoconjugates. *Science*, **261**, 212-215.

WATERFIELD, M.D., MAYES, E.L., STROOBANT, P., BENNET, P.L.P., YOUNG, S., GOODFELLOW, P.N., BANTING, G.S. & OZANNE, B. (1982). A monoclonal antibody to the human epidermal growth factor receptor. *J. Cell. Biochem.*, **20**, 149-161.

WOLTJER, R.L., LUKAS, T.J. & STAROS, J.V. (1992). Direct identification of residues of the epidermal growth factor receptor in close proximity to the amino terminus of bound epidermal growth factor. *Proc. Natl Acad. Sci. USA*, **89**, 7801-7805.